# Empathy as a learning objective in medical education: using phenomenology of learning theory to explore medical students’ learning processes

**DOI:** 10.1186/s12909-022-03696-x

**Published:** 2022-08-19

**Authors:** Elisabeth Assing Hvidt, Anne Ulsø, Cecilie Valentin Thorngreen, Jens Søndergaard, Christina Maar Andersen

**Affiliations:** 1 Research Unit of General Practice, Department of Public Health, University of Southern Denmark, J.B. Winsløwsvej 9 A, 5000 Odense, Denmark; 2 Department of Psychology, University of Southern Denmark, Odense, Denmark; 3 Steno Diabetes Center Odense, Odense University Hospital, Odense, Denmark

**Keywords:** Empathy, Medical education, Denmark, Phenomenology of learning, Qualitative methods

## Abstract

**Background:**

Clinical empathy has been associated with a range of positive patient- and clinician outcomes. Educating medical students to become empathic physicians has in recent years become a clearly pronounced learning objective in medical education in many countries worldwide. Research knowledge about how medical students experience the learning processes conveyed by empathy-enhancing educational interventions is lacking. Our study aimed to explore Danish medical students’ perspectives on which experiences allowed learning processes to take place in relation to empathy and empathic communication with patients.

**Methods:**

We conducted a qualitative research study, involving semi-structured interviews with twenty-three Danish medical students across years of curriculum and universities. Braun and Clarke’s reflexive thematic analysis (RTA) guided the analytical process, moving on a continuum from inductive to deductive, theoretical approaches. Key concepts in regard to learning processes deriving from Amadeo Giorgi’s learning theory were applied to analyse the data.

**Results:**

Learning processes in relation to clinical empathy occured: 1. when theoretical knowledge about empathy became embodied and contextualied within a clinical context 2. through interpersonal interactions, e.g., with peers, faculty members and clinicians, that conveyed behavior-mobilizing positive and negative affect and 3. when new learning discoveries in 2. and 3. were appropriated as a personalized and adequate behavior that transcends the situational level.

**Conclusion:**

Rather than being an immediate product of knowledge transmission, skill acquisition or training, learning clinical empathy is experienced as a dynamic, temporal process embedded in a daily clinical lifeworld of becoming an increasingly human professional.

## Background

 Educating medical students to become empathic physicians has in recent years become a clearly pronounced learning objective in medical education in many countries in the world, reflecting research findings showing that empathy in the context of care and treatment has wide-ranging positive effects for both physicians and patients [1, 2]. So-called clinical empathy, including a predominantly cognitive aspect, is defined in the medical literature as “the ability to understand a patient’s suffering and concerns combined with an ability to communicate this understanding and an intention to help” [3–5]. Clinical empathy has been shown to improve information transfer, diagnostic processes, patient satisfaction and -adherence and thus to increase treatment effectiveness and quality [6–8]. It has also been suggested that empathy protects clinicians from burnout and stress [9], from making medical errors and from malpractice complaints [10]. Moreover, studies show that high scores on empathy among medical students are associated with increased satisfaction with their education, lower levels of stress and burnout, higher ratings of overall clinical competences given by medical education faculty, better interpretational skills assessed by patients and greater teamwork skills [11, 12].

A large body of quantitative research studies have investigated development in self-reported empathy among medical students, some of which show that clinical empathy declines during the medical education [13–15]. Consequently, several educational initiatives to support the professional development of patient-centred and humanistic behaviours, such as communicating in an empathic and compassionate way, have been developed and implemented in the formal medical curriculum [16, 17]. These educational initiatives are varied and comprise literature courses, reflective writing seminars, theatrical performances, student hospitalization experiences, accompanying and assisting patients, communication skill lectures and -workshops [18, 19]. Knowledge dissemination about empathy and empathic communication is built on various learning approaches that altogether focus on reflection and self-reflection [7, 17, 20] via the format of “the traditional lecture” and more practice-based interventions such as role-play and simulation. Whether these strategies are effective for the individual medical student such that empathy can in fact be learned and maintained throughout one’s formation as a physician remains inconclusive, mainly due to study limitations and, on a more abstract and philosofhical level, to disagreement regarding the adequate understanding of empathy [19, 21, 22]. Researchers in the field have questioned whether empathy can in fact be learned as part of the formal curriculum, arguing that looking at empathy as yet another skill to master, in line with history-taking and clinical examinations, turns the highly complex and multidimensional concept of empathy into a caricature that mirrors the natural sciences’ ideal of detachment and objectivity [19, 22].

A Swedish study [23], analysing medical students’ reflective writing pieces on their own empathic development, concluded that together with positive role models, educational activities focusing on reflection and self-awareness could be successful empathy-preserving strategies. Although results from reviews of the literature on empathy-enhancing educational interventions in undergraduate medical education suggest that increasing empathy can be attributed to the above-mentioned interventions [1, 7, 24–26], the teaching and learning of empathy in medical education remains a debated and unresolved issue. In particular, knowledge about how medical students experience the learning processes conveyed by empathy-enhancing educational interventions is lacking, including the developmental processes of understanding, sense-making and behavioral change that students may undergo. A phenomenological approach is particularly relevant in this context focusing as it does on individuals’ subjective experiences of phenomena in their daily lifeworld [27].

Exploring how medical students learn empathy in the course of their educational years from a phenomenological approach might shed light on the various ways in which the phenomenon “learning empathy in medical education” manifests itself in the students’ lifeworld and lived experiences. Drawing on psychologist Amadeo Giorgi’s [27, 28] phenomenological, psychological research on learning, we thus aim to describe the phenomenon of learning empathy in medical education, focusing on the learning processes that medical students experience and to uncover common essences and essential structures of the phenomenon “learning empathy in medical education”. In particular we wanted to investigate: What are medical students’ perspectives on which experiences allowed learning processes to take place in relation to empathy and empathic communication with patients? This question is important to investigate since knowledge hereof can lead to refined communication to students about anticipated learning processes resulting from empathy-enhancing educational interventions and inspire medical educators to further reflect on how to best plan educational interventions that have empathy as a learning objective. In the following, the theoretical framework used as analytical tool is introduced shortly and will be further unfolded throughout the analysis section and thus tied closely to the empirical material.

## Giorgi’s phenomenology of learning

Learning has been a topic of interest in phenomenological circles since the phenomenologists Martin Heidegger and Maurice Merleau-Ponty [29]. Approaching the phenomenon of learning as it is lived and experienced within the lifeworld of day-to-day affairs, allows the phenomenon of learning to show itself unbiased and on its own terms. We draw theoretically on the systematic efforts of the phenomenological psychological researcher Amadeo Giorgi who has described the phenomenon of learning, and the meanings inherent to learning, at a general level [27, 28]. Giorgi has investigated learning by asking subjects to retrospectively describe situations in their everyday life, i.e., ordinary social situations such as learning to drive a car or learning interior decorating, that they defined as experiences of learning [30]. With the help of free imaginative variation, a mental technique through which one alters features of an experience in order to view the phenomenon under investigation from varying perspectives, psychological insights within these experiences are expressed and general essential features of learning as experientially understood, are identified. Giorgi finds that learning includes the following structure: 1. discrimination of relevant parts, 2. the selective functioning of the parts in safe conditions supported by “significant others” and 3. the performance of the parts in normal conditions moving beyond the situational level.

For learning to take place, the learner has to discover false assumptions in relation to the phenomenon of learning, incorrect behavior, etc., thus learning has to be “new” in respect to the subject’s experiential history such that a behavioral possibility exists for the subject that did not exist before. The awareness of a possibility to perform more adequately and the subsequent accomplishment of these possibilities via behavioral change creates, according to Giorgi, the totality of the learning process [30].

Elsewhere, Giorgi has described learning as consisting of knowledge acquisition and subsequent application [27]. Giorgi emphasizes that mere knowledge acquisition in regard to a phenomenon is not “real learning”, supporting the above features that parts perceived as relevant to the individual with his/her history, biography and preunderstandings need to be identified by the learner before learning can take place [27]. This calls attention to the fact that information does not guarantee transformation. The relevant parts need to be experienced bodily and emotionally by the learner followed by a process in which the relevant parts will be applied through a certain behavior manifesting, when successfully applied, a new attitude of perceiving situations in the world.

Another essential feature of learning, according to Giorgi, is that the whole discovery and implementation process of the “new” requires a significant amount of time. Thus learning is temporal – it includes different phases and transition points [27].

## Methods

### Study context

This qualitative study is part of a large study investigating the development of empathy among medical students in Denmark. A mixed-methods methodology was employed in order to measure students’ self-reported empathy level across educational years, measured through the Jefferson Scale of Empathy – Student version [3], and explore students’ individual experiences and perceptions in relation to empathy development. For more information on the large study, see [31].

### Educational context

Medical education in Denmark is university-based, standardized to last 6 years and divided into a three-year bachelor medical education (BME) and a three-year graduate medical education (GME). Differences across the medical educations in Denmark exist in regard to how early students have clinical exposure and patient contact. Generally, the BME comprises basic biomedical science courses in the pre-clinical years (1^st^ and 2^nd^ year) and clinical clerkships towards the end of the bachelor (3^rd^ year), which continue in the GME with an increase in clinical student participation. In some of the medical educations, the curricula foster exposure of medical students to citizens/patients as early as the first year of medical college, either in a social (the home) or clinical setting (e.g., general practice clinics). Clinical exposure, albeit with a low degree of student participation, as part of the bachelor curriculum reflects the overall ambition of contextualising the theoretical teaching that traditionally predominates the early educational years.

### Empathy-related teaching content

The teaching content- and volume of the empathy-related teaching vary slightly across the medical educations in Denmark. A review of the syllabi of the four Danish medical educations show that empathy-related teaching content is primarily placed at the BME comprising subjects like health psychology, philosophy of science, narrative medicine, bio-ethics, etc., some of which are offered as electives. Communication teaching -and training is generally placed towards the end of the BME, encompassing theoretical input and practical training through simulation, continuing as pre-clinical preparation and post-clinical supervision through the GME.

### Recruitment

An announcement summarizing the study and inviting medical students to participate was posted on university web sites. We did not set up any inclusion/exclusion criteria, e.g., demographic variables, but used a convenience sampling [32]. All students who responded to the invitation were forwarded an information letter, detailing the objectives of the study, what it entailed to participate in an interview and information on the collection and processing of the data (complying with the General Data Protection Regulation (GDPR)). All students gave verbal and written consent and were informed that participation in the study was voluntary.

### Participants and data collection methods

The medical students who participated in this study came from all four universities in Denmark. Twenty-three medical students across years of curriculum and universities participated (see Table 1 for an overview of participants). The majority of students were from the University of Southern Denmark (SDU) probably due to local advertisement of the research study.

**Table 1:** Overview of participants from the University of Sourthern Denmark (SDU), Aarhus University (AU), the University of Copenhagen (KU) and Aalborg University (AAU)

Interviews	Number of participants	Gender		University				Year	
Students	23	Female 16	Male 7	SDU 13	AU 4	KU 5	AAU 1	1.-3. 7	4.-6. 16

The interviews were conducted from December 2020 to March 2021 by EAH, a senior qualitative researcher with a background in humanistic health research, and CVT, a junior researcher and psychologist. Because of the corona pandemic, and lockdown periods, all interviews were conducted via video (Zoom) and lasted approximately 60 min. We did not find that conducting the interviews via video compromised the quality of the data, e.g., through technical challenges, lacking rapport building and/or attention to interaction dynamics [33]. Students expressed that they were used to interacting through online platforms due to online lectures and daily social interaction. We were following a semi-structured interview guide to which adjustments were made during the period of data collection, as students’ narratives led to a further contextualization of some of the interview questions. Examples of interview questions are: Could you describe how empathy is addressed as part of your education? Which educational processes do you perceive to enhance and inhibit learning empathy? In what way might your personal empathy have changed during these processes? (see Table 2: Interview guide). Interviews were audio-recorded and transcribed verbatim by a student assistant concurrent with the data collection.

**Table 2:** Interview guide: Empathy in the medical education

**Introductory questions:**
- Which university are you from?
- Which semester and year?
- Why did you choose to study medicine?
**Theme 1: Understanding of empathy**
*To begin with, I would like to ask you some questions relating to your understanding of what empathy is as a general concept, turning then to how you see empathy expressed in a clinical context*
- How would you describe/define empathy as a concept? (e.g., an ability (something one is born with), a skill that can be taught/learned, something that arises in the situation? To feel/fusion with other people’s feelings (sympathy) or to imagine how the other person feels (empathy))?
- According to you, how is empathy displayed in the clinic (e.g., in the meeting between health care provider-patient/patient relatives)?
- Do you think that there can be different ways of showing empathy? If yes, what might these look like?
**Theme 2: Empathy in the medical educational context**
*Now I am interested in hearing more about which role empathy plays in your educational context and how you view the relationship between your education and empathy development among students*
- In your experience, how is empathy adressed as part of your education – e.g., by faculty, peers and clinicians? (e.g., could you describe how the humanities subjects/empathy-enhancing subjects are integrated into your medical curriculum?)
- According to you, should empathy have more or less attention in the educational context?
- Which educational processes do you perceive to enhance and/or inhibit learning empathy and patient-centred, empathic communication?
- In what way might your personal empathy have changed during your education?
**Theme 3: The perceived personal and clinical consequences of empathy**
*Now I am interested in hearing your opinion on what empathy might lead to on a personal and clinical level*
- Could you describe what you perceive as positive/negative consequences for both the physician and patient of empathy?
- What are your thoughts on the relationship between empathy, stress and/or burnout?
- Do you think that you are/will be able to self-regulate your empathy in order to maintain a good mental health as a student/physician?
**Closing comments:**
*Do you have any other comments to the theme of today that you would like to mention?*

### Analysis

The data in this paper consists of retrospective descriptions by medical students on when and how learning was experienced to take place in regard to clinical empathy. Braun and Clarke’s reflexive thematic analysis (RTA) [34] was used to analyze the data through the following steps: initially, transcripts were read by EAH and CVT, taking notes along the way. In a next step, EAH and CVT performed an open and inductive coding in NVivo software (version 12), developing codes that were grounded in the data. Codes were discussed and compared through weekly meetings. A third analyst and junior researcher, AU, with a background in nursing and health sciences, read all the transcripts subsequently. AU was then introduced to all the codes developed by EAH. According to RTA [34], the analytic process is a continuum from inductive to deductive. In accordance herewith, Giorgi’s phenomenology of learning theory was found to provide a description of general learning structures that was helpful in organising the medical students’ situational descriptions of when and how their learning relating to clinical empathy evolved and was transformed. The open codes were revisited and revised iteratively, and Giorgi’s insights apllied to the data. Codes with relevance to Giorgi’s types of learning experiences were selected, adapted and synthesized, seeking to arrive at a general description, across codes, of learning experiences and their general structures (see Table 3).

**Table 3:** Overview of codes and themes

Codes	Themes
Students’ perception of the emphatic focus Maturation process Teaching empathy at the university Narrative medicine Communication training Not realistic Health psychology Not enough or good enough Too theoretical Humanities teachers Lack of contextualization Understanding empathy through the meeting/relationship Understand empathy via clinic Linking theory and practice Sympathy—empathy Development in empathy	*Experiencing learning through discrimination of relevant parts *via* contextualization and application*
Study group Empathy demonstration Empathic teachers Empathy helps create a good culture How empathy is addressed Role models Demonstration of empathy X-factor Putting up on a pedestal Affect Hidden influences Overcompensation Narrative medicine Humanities teachers	*Experiencing learning as an interpersonal, affective process*
Getting better at empathy Development in empathy Authenticity Clinical empathy	*Appropriating the new and the awareness of adequate performance*

In the following, we present through three themes students’ learning experiences- and processes through the analytical lense of Giorgi’s learning theory.

## Results

### Theme 1: Experiencing learning through discrimination of relevant parts via contextualization and application

When reflecting in retrospect on their learning experiences in regard to empathy-related content the students described a preclinical phase (1^st^ and 2^nd^ year of curriculum), in which the degree of learning was perceived as insufficient, and a subsequent clinical phase (3^rd^ to 6^th^ year of curriculum), in which relevant parts of the knowledge transmitted during the pre-clinical phase were suddenly discriminated through the power of contextualization. Six descriptions (#1–6) are presented below, sharing the same essential features.

As expressed by the individual students in the below descriptions #1–3, courses relating to empathy and communication in the preclinical phase at first seemed “annoying” (#1), taking place in “such a fake setting” (#2) where “weird theories were thrown at us” (#3) lacking contextualization:

#1: When you’re in the middle of it [the humanities subjects] it seems quite annoying. And it’s not until later in the process that I kind of get it. At least, at det undergraduate level, we don’t spend a lot of time in the clinic. It’s much later that we get patient contact. And it’s not until then that you realize that imagining yourself in other peoples’ shoes – it’s quite useful. (Female student 5, 5^th^ year)

#2: The communication training just takes place in such a fake setting. Because you’re in a situation where you’re sitting with an actor or whoever…. And there’s plenty of time, and it’s a quiet environment, and you sit and talk for 15 minutes or whatever. And it’s completely different when you’re out there and people are counting on you, and someone is waiting. (Male student 11, 6^th^ year)

#3: We attended a health psychology course right at the beginning of the bachelor, but it was a lousy course, I think. We just had a bunch of weird theories thrown at us and it wasn’t that useful because they weren’t really being contextualized. Furthermore, it was also at an early stage in our studies where we hadn’t yet had so much patient contact. (Male student 21, 6^th^ year)

It is not until meeting the real patients who have the diseases described in the book – and in a setting where “people are counting on you, and someone is waiting” (#2) – that real learning takes place: one suddenly realizes why “imagining yourself in other peoples’ shoes” is useful (#1) being able to make the “link between theory and practice” as stated in the following quote (#4):

#4: It’s not until you meet the patient who has the disease described in the book that you can make the link between theory and practice because you can put a human face on the disease. You remember that the first time I saw a patient with a blood clot in the heart, it was this patient, he looked like this and he felt like this... Then you remember the theory better, but you also remember the more emotional aspect of it. You can better understand the next patients you meet who have the same disease. (Male student 20, 6^th^ year)

According to Giorgi’s phenomenological learning theory, when a person learns, he or she develops a bodily sensitivtity since one’s body is the medium for making sense and figuring things out [35]. This “new” embodied discovery entails a behavioral and experiential possibility that can be applied in the future. In the above description #4, linking theory with practice occurs through the embodied, emotional experience of meeting for the first time a patient suffering from a specific disease (“blood clot in the heart”). The feelings connected hereto are remembered, creating the basis for future empathy towards patients suffering from the same condition. Likewise, in the below description #5 the student describes how she in a situation of potential emotional over-involvement with a patient suddenly understood the difference between “empathy” and “sympathy”, in this medical educational context understood as the difference between emotional overinvolvement that might lead to stress and burnout, and the mostly cognitive ability to understand the patient’s feelings and needs while at the same time avoiding to self-identify [1].

#5: As I recall, it was some humanities teachers who taught us about empathy. And I think, for me at least, it was really hard to relate to. Because it was very theoretical …”this is the definition of empathy”. “This is the definition of sympathy.” I didn’t do well in the humanities subjects [in high school], so for me it was like “I can’t understand this concept”. Or “I just can’t learn it”. And I think it wasn’t until I found myself in the middle of a situation where I had to show empathy towards a patient, feeling the need to shake off the feeling afterwards, that I understood the difference. (Female student 9, 4^th^ year)

The description in #5 also provides historical and biographical context to the other students’ descriptions of insufficient pre-clinical learning processes since it emphazises Giorgi’s point that learning occurs in a relationship between the specific learning content and the person’s experiential history and that “learning cannot be reduced to a specific content, but each content must be understood in terms of the biography or history of the individual” [27].

For the student in description #5, along with many others, humanistic, theoretical content transmitted by humanities teachers, is perceived as “really hard to relate to”, representing a lifeworld that has been deliberately deselected with the choice of enrolling for the medical education. On a specific level, historicity is present because the student reveals her previous experiences with “not doing well in the humanities subjects” in high school, a preunderstanding, possibly embodied, that she uses in the interpretation of the teaching situation as minimally meaningful. Thus, experiencing learning through discrimination of relevant parts via contextualization as expressed by the students cannot be separated from the students’ subjective histories, biographies and thus preunderstandings and bodily sensations, as stressed by Giorgi [27].

Other students, reflecting on learning processes from a post-learning perspective mentioned how, with time and after contact with the clinic, it was experienced as useful that theoretical concepts and “boxes” had been presented to their consciousness in the preclinical learning phase:

#6: I’ve learned about it [empathy], through my work. I mean, where you learn that there is a psychosocial influence, and there are also some relatives who would like to be included. And you have learned about some fine concepts and “boxes” through narrative medicine that you can use later. (Female student 8, 4^th^ year).

### Theme 2: Experiencing learning as an interpersonal, affective process

While speaking of powerful contextualizing experiences, clear descriptions emerged of learning processes taking place in an interpersonal, affective contexts. Giorgi talks about learning as a “radically inter-human phenomenon” [27] and about the facilitative role of another person, a “significant other”, to be a key factor in the experience of learning when it occurs positively [36]. In a similar way, the students in this study described learning empathy as a relational process and highlighted the positive effect on their own learning of empathy demonstrations by people, that they could identify themselves with: peers, faculty members and clinicians. Eight descriptions are presented below that all share the same essential features in regard to learning empathy, i.e., students observe (and live) situations in which other peoples’ behavior, values and attitudes towards patients, create emotions and moods that motivate them for a certain empathic behavior and future professional identity.

In description #7 the student explains how she feels that her empathy has increased during her study years because of her peers in the study group, who are “incredibly nice people”. Their behavior is “contagious”, and observing the positive effect that it appears to have on other people, makes her inclined to reproduce their kindness:

#7: In fact, I think the people in my study group, they’ve helped me increase my empathy. Because they’re incredibly nice people. And then you just go like: “Well, it would be very nice to be a nice person”. Because their way of being, it also rubs off on you, and you see that it has a positive effect. And then you just become inclined to do what they do. (Female student 4, 5^th^ year)

The student furthermore exemplifies, in the following description #8, how faculty members’ demonstrations of an empathic behavior in a clinical situation, e.g., breast palpation, are valued and efficient learning situations:

#8: A good example is one of the teachers we had in breast cancer, who said that the patients prefer that we don’t wear gloves, because it creates a distance. And she demonstrated ways to create presence and trust. I mean, that way you focus on empathy. The more empathic your teachers are, the more you’ll come to think about it later. Because if they were all like “patients are stupid; they’re ass holes,” then this is the attitude that you yourself will develop. (Female student 4, 5^th^ year)

In description #9–11, the students emphazise the point relating to the interpersonal, affective context of learning: when observing how empathy is embodied, enacted and linguistically situated by experienced physician and -nurse role models in the clinic, feeling an alignment in personal values, they feel “really inspired” (#11), emotionally enriched (“…they touch me deeply”, #11) and learning takes place (“you embrace their behavior and actions”, #10).

#9: Well, you meet role models and think, “really cool”. And I learn something from most doctors I meet out there by just observing them. And you always benefit a lot from observing other people’s communication and interaction, thinking this was a good way to ask that question. Or: “really good body language, good contact with the patient”. So, one always learns something from observing others, I think. (Female student 14, 4^th^ year)

#10: I have personally experienced on my own body what happens when you have role models in front of you - when faced with doctors who are in fact damn good at their work. Well, they're the ones you're supposed to be mirroring. You must embrace their behavior and actions. And this is only possible in the clinic. (Female student 15, 1st year)

#11: It’s not all the biomedical stuff going on in the clinic that I learn most from, but the human. The personal. The doctors who are really positive role models from whom I feel that I can learn a lot, observing how they work, following them around. And in this empathy plays a central role. And I find it really inspiring. I really feel like I can learn from that. Because, the biomedical stuff, I can learn from the books and we will learn it along the way, but those who manage to create this contact and who in fact show empathy, they touch me deeply. It’s so amazing to see and experience. (Female student 23, 6^th^ year)

In all of the above descriptions, skills can be observed for future rehearsing in a safe learning environment and attitudes towards different groups of patients, ethical aspects of care and students’ own role and identity as a doctor, can be developed.

Learning situations would also occur that provoked negative affect in students, e.g., injustice, anger, frustration, social indignation. Desriptions #12–14 exemplify this: the situations start with the perception of a problematic conduct, attitude or disalignment of values that provoke negative affect and inspire students towards future behaviors.

#12: We’ve had one teacher in communication training, who was like “you are medical students, so you’re all from the same privileged background, and that's just the way it is, period.” And “You can’t understand people who are socially deprived. And you have to learn to accept this” and things like that. Well, that attitude, I was just like,” You don't know anything about that”. I mean, people have a lot of different backgrounds in here, so don’t talk like that! I really felt like we were all being categorized, thinking: “you don't know anything about that! And also that thing about, “every patient you’re going to meet will simply be at a lower level than you”. (Female, student 5, 5^th^ year)

In #12 the student describes the negative affect that arises in her when a teacher categorizes medical students in general as belonging to the priviledged class, and therefore as having to accept the unbridgeable divide between themselves and their patients in future communication scenarios. The anger that arises in her (“I really felt like we were all being categorized, thinking: “you don’t know anything about that!”) makes her motivated to correct inadequate assumptions through own behavior towards patients. This reactive learning process is also seen in description #13 when a student’s observations of the repulsive behavior of her peers towards patients provokes the feeling in her that “I must be ready to compensate”:

#13: When I see future doctors with an unpleasant attitude, then I feel that I must be ready to compensate. Then I need to be even nicer because somebody is being an ass hole. I’ve just experienced it the other day: I work in a corona test center, and I have a colleague, he’s a real ass hole. Like to an extreme extent. But he’s working there, and he’s a medical student, and he will probably become a doctor once. I hope not. And I can feel that when I'm at work with him, you're really kind of aware that the people who meet him, will not walk away feeling that we’re all like that. So, you must give more of yourself, and then you get exhausted. And I know that a lot of us do this at work. We’ve been talking about it many times. That a lot of us do this. It’s so exhausting. (Female student 5, 5^th^ year)

The importance of being able to relate to ones’ role models for learning to take place and learning as “an interhuman phenomenon” is further emphasized in the following descriptions #14 and #15 where the students create a link between unsuccessful learning in relation to empathy and experiences of lacking relatability between teachers and students.

#14: Well, I think it was wrong to choose humanities teachers instead of choosing teachers who are within our profession. Because it made it very difficult for them to relate to us, and for us to relate to them. Because, if you’re going to learn about empathy, it’s kind of a prerequisite that you learn to understand each other, and then it’s kind of strange to have someone who doesn’t understand our situation at all, and we don’t understand them at all. (Female student 7, 5^th^ year)

#15: In some of the courses those who have been teaching us were humanities teachers and I have sensed that some are setting up a front against it, saying that they can’t see the point with it and then they decline taking part in the discussion that they try to create. (Female student 5, 5^th^ year)

To sum up, the students’ descriptions demonstrate how learning occurs in an interpersonal, affective context in which other peoples’ attitudes, values and behaviors are powerful learning motivators. Learning occurs as a reaction to a desired behavior that one can relate to and feels an urge to reproduce, or as a reaction against an undesired, repelling behavior, at times paradoxically strengthening the resolve to be compassionate and empathic. As a result, learning derives from a personally experienced value alignment- or conflict, clarifying who one wants to be as a future professional.

### Theme 3: Appropriating the new and the awareness of adequate performance

As seen in theme 1 and 2, learning empathy takes place through discrimination of relevant parts through contextualization and through interpersonal interactions that convey behavior-mobilizing affect. In these two phases, however, the new phenomenon of clinical empathy, is still not personalized and the adequate behaviors not entirely lived. According to Giorgi, the first learning phases still constitute partial learning that is likely to continue with more experience and appropriation of the new. As the learning comes to be adequately performed in real life conditions it takes on a personal sense of familiarity that transcends the specific situation in which it was experienced [36]. Full learning is ultimately defined as a new way of looking and perceiving [27]. The type of learning that we have called “appropriating the new and the awareness of adequate performance” is based on 3 descriptions connected to students’ longer periods of residency. What makes these descriptions examples of full learning is that they show how practicing one’s behaviour in encounters with patients of multiple backgrounds leads to an awareness of behavioral competence that goes beyond the situational level. In description #16 a student in her last educational year describes how showing empathy towards all patients “with time and practice becomes easier”. From being a depersonalized learning task that one must remind oneself of performing on a situational level, empathy becomes a “standard response” and “a baseline empathy with which you meet people”.

#16: I think that it’s getting easier [to show empathy], especially following a hospital stay for example, where one must interact with patients and their relatives. And we often interact with people whom you wouldn’t otherwise have met, or befriended, or have forged a relationship with. I have experienced that showing empathy towards all people in such a context and being empathic to them, becomes easier. So, with time and practice it becomes easier, and I also think that it becomes a natural response to people and not just something you choose, or where you think: “now I must also remember to be empathic” or “an empathic thing to do right now would be to comfort or...”. It's turning into a standard response, yes. It becomes a “baseline empathy” with which you meet people. (Female student 18, 6^th^ year)

#17: My understanding of what empathy is has certainly developed. When I started at the medical education, empathy was... Yes, it’s kind of difficult to explain, but it has probably developed into being a more inclusive kind of empathy. It’s not some kind of cape, that I’m putting on, so that when wearing it now, I’m empathic... it’s an inclusiveness, which is much more about the person in front of me. And a lot less about myself, really. That I must be empathic. But it’s about what people need in terms of empathic behavior. I didn’t have that angle in the beginning, I think. (Female student 18, 6^th^ year).

In description #17 empathy is no longer “some kind of cape that I’m putting on”, but an attitude of “inclusiveness” towards other people, reflecting a wish to meet patients’ individual needs for empathy.

In description #18 learning empathy, and “knowing how to act towards patients who have these and these feelings” is also experienced to take place through continuous interaction- and communication with patients in the clinic. The student has reached a point in her/his learning process in which she/he feels that she/he has come to master an empathic response to patients – thus feeling confident in her/himself. This brings a certain finality to the situation: for her/him, being empathic towards patients has increasingly become a professional response in clinical situations and a natural part of a performance that she/he needs no more be aware of.

#18: Well, I think, the fact that I interact with a lot of people makes me better at it [showing empathy], and that I see a lot of patients with whom I keep communicating. Because, in some way or another, you practice all the time and get better at it. Because it’s a professional kind of empathy, as I like to call it. That you know how to act towards a patient who has these and these feelings. I think that the first time you must give a harsh message to relatives, for example, you are not good at it, and then you get better and better. (Male student 10, 5^th^ year)

All three descriptions demonstrate that full learning is a function of the learner’s own continually unfolding involvement with the clinical lifeworld context and therefore at the same time a process that makes the student more confidently her/himself as a future physician.

## Discussion

This study has investigated medical students’ perspectives on which experiences allowed learning processes to take place in relation to empathy and empathic communication within a medical educational context, providing evidence that learning clinical empathy has transition points that define phases and levels of embodied learning that can be outlined in general terms as follows:

1. Learning empathy in the context of medical education emerges when theoretical knowledge about empathy meets the force of a clinical lifeworld context in which relevance and meaningfulness of the acquired knowledge is discriminated and unfolded. This unfolding of new, integrated meaning opens behavioral possibilities that can be applied in the future and form part of one’s future professional identity. 2. Learning empathy proceeds within fields of interaction. Other peoples’ (significant others’) attitudes, values, professional backgrounds, verbal and non-verbal conducts are essential constituents of an emotional and affective learning process, mobilizing own future behavior. The students learn empathy by being attentive to significant others, then applying for themselves that knowledge. 3. As the new learning discoveries are appropriated, to a high degree on an emotional level, they turn into a personal and embodied response the implications of which transcend specific situations, becoming a new way of perceiving and approaching the clinical lifeworld as future empathic physician from thenceforward.

In light of this three-fold structure, a phenomenological view of learning empathy involes far more than mere intellect. It entails a gradual, dynamic process of becoming increasingly mature and human through interaction with the world and through a synthesization of cognition, affect, body and sociality. In contrast to current medical educational practice, where large amounts of knowledge is transmitted to the student, learning empathy is a holistic process, involving the integrated functioning of the person – thinking, feeling, perceiving and behaving. It also contrasts with biomedicine’s scientific ideals of neutrality and objectivity since it confronts the person with the meaning of one’s own being-in-the-world-with-others. On the basis of our phenomenologically-informed analysis, we suggest the usefulness of presenting students and faculty with a phenomenological view of the essential structures and features of learning empathy within a medical educational context to support teachers’ and students’ understanding of the learning processes. Within the first theme, we have given insight into how lacking contextualization of learning content can be problematic for students, and for their learning processes. We therefore believe that early contextualization of theoretical knowledge about empathy and empathic communication needs to be worked at in the educational planning of empathy-related teaching. The planning phases should also include reflections on whether the teaching models employed respond to perceived needs of the students. For example, our findings indicate that students realize the relevance of empathy-enhancing teaching when presented in clinical demonstrations and when taught by teachers with clinical backgrounds to which they can relate. That learning is best accomplished through authentic practice-based experiences within a “real” clinical environment, described in themes 2 and 3, is a key point in the medical educational theory called experience based learning (ExBL), developed by professor of medical education, Tim Dornan [37–39]. Drawing on insights from social constructivist learning theory [37], and experiential learning theory [40], Dornan emphazises that medical students’ capability, both intellectual, practical and affective (including the capability to provide empathic care), and professional identity formation, depends on learning processes taking place through participation within clinical practice settings in which supporting clinicians share their expertise with students. As such, ExBL is not about “teaching” but about “supported learning” through participation experiences facilitated by clinicians in practice.

The successful learning processes- and outcomes deriving from observing other peoples’ behavior, also emphazised in theme 2 and 3, is furthermore widely accepted as a highly influential learning method described in detail through the behaviorist learning theoretician Albert Bandura’s social learning and role model theory [41]. To enhance successful learning processes of clinical empathy founded on observational learning in clinical settings, a more conscious and consistent approach involving clinical teachers well equipped for the empathy teaching task is needed [42]. For such clinically-based didactic strategies to be realized university- and hospital institutions need to rethink their priorities (and above them politicians operating on a macro-level) both in relation to which teaching content is prioritized in the clinic (biomedical medicine or communication) and thus the amount of monetary- and time resources spent on clinician supervision training.

### Strenghts and limitations

To our knowledge, our study is the first to explore from a phenomenological perspective medical students’ experiences with the phenomenon “learning empathy in medical education”. This has led us to identify essential features of the said phenomenon that goes beyond a mere situational level. Thus, despite being limited to a Danish medical educational context, the analysis taking place at a general level of explication (aiming at finding general characteristics- and structures of the phenomenon at hand) increases the transferability of the results to other educational settings with similar curricular. Another strength in the study is that the analysis was conducted in collaborations between three researchers with different professional backgrounds, increasing the trustworthiness of the findings.

However, there are some limitations to our study. We included a convenience sample of students from all four universities in Denmark. Therefore, the number and the gender of the students were not evenly distributed across the four universities which means that the findings primarily reflect learning experiences from students deriving from three out of the four universities. However, being a multi-institutional study, the findings are more transferrable to similar educational contexts than studies made in a single institution.

## Conclusion

Our study demonstrates that students’ descriptions of successful learning processes in relation to clinical empathy goes beyond traditional communication teaching involving knowledge transmission, skill acquisition or training. Learning clinical empathy is to a much higher degree experienced as a dynamic, affective and temporal process embedded in a daily lifeworld of becoming an increasingly mature and human professional. In order to enhance the learning of empathy in a medical educational context, we suggest that attention is given to reinforcing the contextualization- and application dimensions through experience-based learning within clinical settings with supporting clinicians acting as positive role models and providing relevant feed-back to students so as to minimize feelings of irrelevance and non-relatability among junior students.

## Data Availability

There is no public availability to the interview transcripts outside of the research team due to reasons of confidentiality. Data are however available from the corresponding author on reasonable request.

## References

[1] Stepien KA, Baernstein A (2006). Educating for empathy. A review. J Gen Intern Med.

[2] Light A, Gupta T, Burrows A, Nandakumar M, Daniel A, Karthikeyan S (2019). Learning empathy: the medical student perspective. Clin Teach.

[3] Hojat M, Gonnella JS (2015). Eleven Years of Data on the Jefferson Scale of Empathy-Medical Student Version (JSE-S): Proxy Norm Data and Tentative Cutoff Scores. Med Princ Pract.

[4] Derksen F, Bensing J, Lagro-Janssen A (2013). Effectiveness of empathy in general practice: a systematic review. Br J Gen Pract.

[5] Mercer SW, Reynolds WJ (2002). Empathy and quality of care. Br J Gen Pract.

[6] Neumann M, Bensing J, Mercer S, Ernstmann N, Ommen O, Pfaff H (2008). Analyzing the “nature” and “specific effectiveness” of clinical empathy: A theoretical overview and contribution towards a theory-based research agenda. Patient Educ Couns.

[7] Costa-Drolon E, Verneuil L, Manolios E, Revah-Levy A, Sibeoni J (2021). Medical Students’ Perspectives on Empathy: A Systematic Review and Metasynthesis. Acad Med.

[8] Decety J, Fotopoulou A (2015). Why empathy has a beneficial impact on others in medicine: unifying theories. Front Behav Neurosci.

[9] Wilkinson H, Whittington R, Perry L, Eames C (2017). Examining the relationship between burnout and empathy in healthcare professionals: A systematic review. Burn Res.

[10] Riess H, Kelley JM, Bailey RW, Dunn EJ, Phillips M (2012). Empathy Training for Resident Physicians: A Randomized Controlled Trial of a Neuroscience-Informed Curriculum. J Gen Intern Med.

[11] Hojat M, Vergare M, Isenberg G, Cohen M, Spandorfer J (2015). Underlying construct of empathy, optimism, and burnout in medical students. Int J Med Educ.

[12] Hojat M, Bianco JA, Mann D, Massello D, Calabrese LH. Overlap between empathy, teamwork and integrative approach to patient care. Med Teach. 2015;37(8):755–8 10.3109/0142159X.2014.971722 25314019

[13] Andersen FA (2020). Johansen A-SB, Søndergaard J, Andersen CM, Assing Hvidt E: Revisiting the trajectory of medical students’ empathy, and impact of gender, specialty preferences and nationality: a systematic review. BMC Med Educ.

[14] Sulzer SH, Feinstein NW, Wendland CL (2016). Assessing empathy development in medical education: a systematic review. Med Educ.

[15] Neumann M, Edelhauser F, Tauschel D, Fischer MR, Wirtz M, Woopen C, Haramati A, Scheffer C (2011). Empathy decline and its reasons: a systematic review of studies with medical students and residents. Acad Med.

[16] Petrou L, Mittelman E, Osibona O, Panahi M, Harvey JM, Patrick YAA, Leedham-Green KE (2021). The role of humanities in the medical curriculum: medical students’ perspectives. BMC Med Educ.

[17] Zhou YC, Tan SR, Tan CGH, Ng MSP, Lim KH, Tan LHE, Ong YT, Cheong CWS, Chin AMC, Chiam M (2021). A systematic scoping review of approaches to teaching and assessing empathy in medicine. BMC Med Educ.

[18] Carr SE, Noya F, Phillips B, Harris A, Scott K, Hooker C, Mavaddat N, Ani-Amponsah M, Vuillermin DM, Reid S (2021). Health Humanities curriculum and evaluation in health professions education: a scoping review. BMC Med Educ.

[19] Halpern J (2014). From idealized clinical empathy to empathic communication in medical care. Med Health Care Philos.

[20] Pohontsch NJ, Stark A, Ehrhardt M, Kötter T, Scherer M (2018). Influences on students' empathy in medical education: an exploratory interview study with medical students in their third and last year. BMC Med Educ.

[21] Kelm Z, Womer J, Walter JK, Feudtner C (2014). Interventions to cultivate physician empathy: a systematic review. BMC Med Educ.

[22] Hooker C (2015). Understanding empathy: why phenomenology and hermeneutics can help medical education and practice. Med Health Care Philos.

[23] Seeberger A, Lönn A, Hult H, Weurlander M, Wernerson A (2020). Can empathy be preserved in medical education?. Int J Med Educ.

[24] Batt-Rawden SA, Chisolm MS, Anton B, Flickinger TE (2013). Teaching empathy to medical students: an updated, systematic review. Acad Med.

[25] Patel S, Pelletier-Bui A, Smith S, Roberts MB, Kilgannon H, Trzeciak S, Roberts BW (2019). Curricula for empathy and compassion training in medical education: A systematic review. PLoS ONE.

[26] Fragkos KC, Crampton PES (2020). The Effectiveness of Teaching Clinical Empathy to Medical Students: A Systematic Review and Meta-Analysis of Randomized Controlled Trials. Acad Med.

[27] Giorgi A, Fischer C, Murray EL (1975). An Application of Phenomenological Method in Psychology. Duquesne Studies in Phenomenological Psychology Volume 2, edn.

[28] Giorgi A, Valle RS, Halling S (1989). Learning and Memory from the Perspective of Phenomenological Psychology. Existential-Phenomenological Perspectives in Psychology.

[29] DeRobertis EM (2017). The phenomenology of learning and becoming: Enthusiasm, creativity, and self-development.

[30] Giorgi A (1985). Phenomenology and psychological research.

[31] Assing Hvidt E, Søndergaard J, Hvidt NC, Wehberg S, Büssing A, Andersen CM (2020). Development in Danish medical students' empathy: study protocol of a cross-sectional and longitudinal mixed-methods study. BMC Med Educ.

[32] Paik LS, Shahani-Denning C, Rogelberg SG (2017). Convenience Sampling. The SAGE Encyclopedia of Industrial and Organizational Psychology.

[33] Carter SM, Shih P, Williams J, Degeling C, Mooney-Somers J (2021). Conducting Qualitative Research Online: Challenges and Solutions. Patient.

[34] Braun V, Clarke V (2021). One size fits all? What counts as quality practice in (reflexive) thematic analysis?. Qual Res Psychol.

[35] Derobertis E. Phenomenology of Learning. In: Derobertis E, editor. The Phenomenology of Learning and Becoming: Enthusiasm, Creativity, and Self-Development. New York: Palgrave Macmillan US; 2017. p.19–40.

[36] Giorgi A. Learning and Memory from the Perspective of Phenomenological Psychology. In: Valle RS, Halling S, editors. Existential-phenomenological perspectives in psychology: Exploring the breadth of human experience. Plenum Press; 1989: 99–112.

[37] Yardley S, Teunissen PW, Dornan T (2012). Experiential learning: AMEE Guide No. 63. Med Teach.

[38] Dornan T, Scherpbier A, Boshuizen H (2009). Supporting medical students workplace learning: experience-based learning (ExBL). Clin Teach.

[39] Dornan T, Conn R, Monaghan H, Kearney G, Gillespie H, Bennett D (2019). Experience Based Learning (ExBL): Clinical teaching for the twenty-first century. Med Teach.

[40] Kolb AY, Kolb DA (2005). Learning Styles and Learning Spaces: Enhancing Experiential Learning in Higher Education. Acad Manag Learn Educ.

[41] Horsburgh J, Ippolito K (2018). A skill to be worked at: using social learning theory to explore the process of learning from role models in clinical settings. BMC Med Educ.

[42] Perron NJ, Sommer J, Hudelson P, Demaurex F, Luthy C, Louis-Simonet M, Nendaz M, De Grave W, Dolmans D, van der Vleuten CP (2009). Clinical supervisors' perceived needs for teaching communication skills in clinical practice. Med Teach.

[43] World Medical Association Declaration of Helsinki (2013). Ethical principles for medical research involving human subjects. JAMA.

